# Multi-dimensional integrated dance therapeutics (MIDT) for fall prevention in community-dwelling older adults

**DOI:** 10.3389/fpubh.2026.1780671

**Published:** 2026-05-29

**Authors:** Cynthia Sau Ting Wu, V Wan Chee Yim, Crista Yan Yan Kwok

**Affiliations:** 1 School of Nursing, The Hong Kong Polytechnic University, Hong Kong, Hong Kong SAR, China; 2 Centre for Community Cultural Development, Hong Kong, Hong Kong SAR, China

**Keywords:** community-dwelling older adults, dance therapeutics, fall prevention, fall risk, multi-dimensional health outcomes, physical, psychological, state of well-being

## Abstract

**Introduction:**

Recent studies advocate for dance interventions that address the physical and psychological factors associated with fall risk. The multi-dimensional integrated dance therapeutics (MIDT) program engages participants in physical, psychological, and environmental interactions within a single dance therapeutics framework, rather than focusing solely on balance, strength, or mobility outcomes; it therefore promotes an integrated approach for fall prevention among community-dwelling older individuals. With the increase in the aging population, there is a growing demand for developing an integrated framework for fall risk reduction in the community.

**Aim:**

This study aims to examine the significant impacts of MIDT on four health outcomes: balance, mobility, fear of falling, and state of well-being.

**Methods:**

A quasi-experimental study without a control group was conducted to examine the effects of the MIDT program on paired dependent groups. Physical health outcomes were assessed using the unipedal stance test (balance) and the Timed Up and Go (TUG; mobility) test. Psychological outcomes, including fear of falling and state of well-being, were measured using the Fear of Falling Questionnaire - Revised (FFQ-R) and the 5-Item World Health Organization Well-Being Index (WHO-5), respectively. The MIDT program consisted of twelve 1.5-h sessions that included cognitive learning of fall and fall prevention concepts, core muscle strengthening exercises, dynamic balance training, body–mind intervention techniques, fall response training, and interaction with the environment.

**Results:**

A sample of 46 older adults was recruited from two community centers from February to May 2024. Significant positive changes were observed in both physical and psychological outcomes.

**Discussion:**

The findings contribute to the theoretical understanding of fall prevention practices. The positive outcomes demonstrate the efficacy of the MIDT program in fall prevention.

**Conclusion:**

This study generates new knowledge and contributes to the development of integrated fall prevention practices for community-dwelling older adults. A multi-dimensional integrated approach incorporating fall response training and interaction with the environment, in addition to strengthening physical outcomes, is recommended.

## Background

According to the World Health Organization (1), the primary fall risk factors include biological, behavioral, social/economic, and environmental factors. “Biological risks are associated with human body changes due to aging such as the decline of physical, cognitive, and affective capacities, and the co-morbidity associated with chronic illnesses. The behavioral risks include those concerning human actions, emotions, and daily choices. Social/economic factors are related to social conditions and economic status of individuals, as well as the capacity of the community to challenge them. These factors include low income, low education, inadequate housing, and lack of social interaction. Environmental factors encapsulate the interplay between individuals’ physical conditions and their surrounding environment, which includes hazards at home and in public spaces.”

Referring to a systematic review and meta-analysis on recurrent falls in older adults, Jehu et al. (2) also identified four key fall risk factors—balance, mobility, psychological, and sensory factors—that are associated with a higher risk of recurrent falls. The need for an integrated, multi-dimensional fall prevention program for community-dwelling older adults is therefore evident.

### Literature review

In a review study on risk assessment and prevention of falls in community-dwelling older adults Colon-Emeric et al. (3) noted that falls result from age-related physiological changes caused by multiple intrinsic and extrinsic risk factors. Gait and balance disorders, orthostatic hypotension, sensory impairment, and environmental hazards are among the major modifiable risk factors in this population. The study reported that functional exercises aimed at improving leg strength and balance are recommended for fall prevention in both average- and high-risk populations. Limited studies on fall-prevention include the environmental factors to reaction and fear of fall towards the state of wellbeings. In studies on global guidelines for fall prevention and management in older adults (4, 5), the authors have emphasized the importance of using multi-dimensional interventions to address multiple risk factors. Furthermore, a systematic review identified a positive association between postural changes and an increased risk of loss of balance and falls (6). Fewer studies have focused on the process of postural adjustment to reduce fall risks. Thomas et al. (7) stated that the majority of interventions target static balance, with fewer focusing on dynamic balance or postural control. Similar to postural changes or adjustments, fall efficacy is important for older adults to reduce their risk of falling (8); however, fewer studies have included specific training aimed at promoting the state of well-being for fall prevention.

Dance can improve balance, gait, dynamic mobility, muscular strength, and overall physical performance, reducing the risk of falls (9). Some studies have also demonstrated that dance shares the holistic features of Tai Chi, combining physical, cognitive, and social elements (10). Dance therapeutics programs have been conceptualized as psychotherapeutic modalities that foster emotional, social, cognitive, and physical integration, with the overarching goal of enhancing health and well-being through dance and body movements (11). The preliminary findings from the pilot phase of the first 8-week basic program (IDDMT), conducted by the authors and their team, also identified significant positive correlations between increases in physical strength and well-being and reductions in fall risk factors (12). Pitluk Barash et al. (23) also investigated how grounding techniques and sensory perception in dance movement may reduce falls and strengthen the sense of security. They further explored with participants how recognizing, understanding, and facilitating their movements and body positions contributed to a sense of security, stability, and body control (22).

Aim: This study aims to examine the significant impact of multi-dimensional integrated dance therapeutics (MIDT) on four health outcomes—balance, mobility, fear of falling, and state of well-being—among community-dwelling older adults.

## Methods and procedures

### Study design and participant recruitment

A quasi-experimental study without a control group was conducted. Participants were invited through phone calls and emails to recruitment sessions at two community health centers, primarily serving older adults. They were recruited according to the selection criteria. Participants were provided with detailed information about the program through information sheets.

### Ethics consideration

Participants were fully informed about the potential risks and benefits of program participation. Informed consent was obtained for using program evaluation data. No identifiable personal.

Information was disclosed in any public report. Participants had the right to withdraw from the evaluation study at any time without any consequences for their participation in the program.

### Selection criteria

The inclusion criteria included participants who had attended the IDDMT basic fall program (12). The advanced program served as a top-up, second phase of the basic fall program. The age criterion was set at 50 years or older, corresponding to the classifications of young-old elders (50–64) and old-old elders (65–79) in the target community.

Participants were able to read Chinese and understand spoken Cantonese for the dance instructions. They were also capable of providing informed consent and attending at least eight sessions, including the first and last sessions for pretest and posttest evaluations.

The exclusion criteria included a history of bone or joint injury and any falls in the past 3 months. Participants were required to complete a self-declaration of physical fitness checklist before beginning the program. Individuals were excluded if they were unable to provide the fitness declaration necessary for participating in the dance exercise.

## Program intervention

### Structure and fidelity of the program

The MIDT program consisted of two phases: An eight-session, 8-week basic program and an eight-session, 8-week advanced program. Each session lasted 1.5 h. The basic program (IDDMT) focused on cognitive learning of fall and fall prevention concepts and dance therapeutics practice targeting foundational physical capacity and psychological awareness. The physical component focused on lower extremity neuromuscular conditioning, static balance stabilization, and musculoskeletal flexibility. The psychosocial component incorporated multisensory stimulation, peer-mediated social interaction, and training in body–mind awareness to promote participant self-regulation. Each session consisted of a 10-min warm-up session, a 25-min physical session, a 25-min psychological activity session, a 25-min dance choreography session, and a 5-min cool-down session.

### Fidelity of the program

The two interventionists adhered strictly to the practice principles. During the pilot phase, the two interventionists would validate each other practice to assure the consistency of the intervention approaches, duration and frequency.

### Program structure

Table 1 shows the program structure.

**Table 1:** The program structure.

Session and duration	Phase I: Basic program (8 weeks)		Phase II: Advanced program (8 weeks)	
Focus	Objectives	Key components	Objectives	Key components
Warm up (10 min)	Getting to know each other	Body game	Greeting each other	Body relaxation/movement
Physical exercises (25 min)	Strengthening physical muscles	Exercises for lower limb strength, balance, and stretching	Strengthening physical muscles	Exercises for core muscle strength, dynamic balance, body centering, and advanced lower limb training
Psychosocial exercises (25 min)	Enhancing psychosocial wellness	Activities related to the five senses, social interaction, and body/emotion awareness	Enhancing psychosocial wellness	Activities for posture control and fall efficacy, environmental perception and interaction, and deepening of sensory, social, and body/emotion awareness
Choregraphy (25 min)	Deepening the process of fall prevention through the integration of physical and psychological elements	Integration of physical exercises into musical and rhythmic movements; parts of the choreography inspired by contemporary dance, an expressive style of dancing that facilitates greater social and emotional expression	Deepening the process of fall prevention through the integration of physical and psychological session elements	Physical and psychological exercises targeting the four risk factors, allowing for deeper maintenance and reinforcement, integrated into the basic class structure
Cool-down session (5 min)	Relaxing tightened muscles	Body stretching with breathing	Relaxing tightened muscles	Body stretching with breathing

The advanced program (MIDT) shifted the focus toward functional complexity. Physical training prioritized core stability, dynamic postural control, and targeted lower limb neuromuscular engagement (incorporating body-centering techniques). The psychological framework emphasized proprioceptive and deeper body–mind awareness, cognitive and body reaction strategies for fall prevention (perceived self-efficacy), and adaptive motor control and mental training within complex environmental contexts.

The group practice sessions focused on dance movements specifically related to fall prevention and combined with body–mind intervention training. Participants were organized into groups of 12 to 15 individuals. The practice sessions progressively incorporated physical strengthening, balance training, fall-related dance movements, and experiential body–mind intervention elements such as body awareness, coordination, and integration. The dance choreography was designed to enhance fall efficacy while incorporating therapeutics elements of body–mind awareness. To encourage adherence, participants were provided with a take-home video for independent practice.

### Data collection and measures

This study employed a pretest–posttest design to evaluate changes over time. At baseline, participants’ characteristics—including demographics, fall history, and relevant health conditions—were recorded. To provide a comprehensive evaluation, a mixed methods approach was used for outcome measurements: Well-being and fear of falling were assessed through self-reported scales, while mobility and balance were measured using objective, performance-based assessments. A total of four distinct measures were utilized to evaluate physical performance and psychological states. (1) Physical balance was measured using the unipedal stance test (balance), which is sensitive to fall risk. Participants were instructed to stand on one leg—starting with the right leg, then the left—with eyes open, and the duration (in seconds) was recorded. (2) Physical mobility was assessed using the Timed Up and Go (TUG) test, measured in seconds(17). The TUG test demonstrates excellent intra-rater, inter-rater, and test–retest reliability and is widely adopted in health service settings(18). (3) Fear of falling was measured using the Fear of Falling Questionnaire – Revised (FFQ-R), which has a Cronbach’s *α* ranging from 0.72 to 0.83 and a test–retest reliability of 0.82. The FFQ-R is a 15-item self-report questionnaire, rated on a 4-point Likert scale from 1 (strongly disagree) to 4 (strongly agree)(16, 21). The total possible score ranges from 15 to 60, with higher scores indicating greater fear of falling. (4) Psychological well-being was assessed using the 5-item World Health Organization Well-Being Index (WHO-5), a short self-report measure with a sensitivity of 0.93 and a specificity of 0.83 for detecting depression across young to older adults (20).

## Data analysis

All statistical analyses were conducted using IBM SPSS Statistics, Version 28.0. The level of statistical significance for all tests was set at a *p*-value of <0.05. Descriptive statistics were used to summarize participant characteristics and health-related variables. Significant differences between pretest and posttest means for balance and mobility were analyzed using dependent samples *t*-tests. For fear of falling and well-being, the Wilcoxon signed-rank test was employed to assess median differences between time points.

## Results

### Demographics

As presented in Table 2, 46 participants were women (88.5%) and six were men (11.5%). The mean age was 66.77 years, with a range of 52–79 years. A total of 18 participants (34.6%) were aged between 50 and 64 years, and 34 participants (65.4%) were aged between 65 and 79 years. Regarding personal income, including government subsidies, 23.1% (*n* = 12) reported zero income, 36.5% (*n* = 19) earned below HK5000, 26.9% (*n* = 14) earned between HK5001 and HK10000, and 11.5% (*n* = 6) earned between HK10001 and HK20000.

**Table 2:** Demographics, health conditions, and history of falls.

Variables	*N* (%)
(Q1) Demographics Gender	
Men	6 (11.5)
Women	46 (88.5)
Age (years)	
50–64	18 (34.6)
65–79	34 (65.4)
Mean age	66.77
Monthly personal income ($HK)	
0	12(23.1)
Below 5,001	19(36.5)
5,001–10,000	14 (26.9)
10,001–15,000	6 (11.5)
Missing	1(1.9)
(Q2) History of falls in the past 12 months	
Yes	13 (25.0)
No	39 (75.0)
(Q3.1) Chronic diseases	
Yes	19 (36.5)
No	33 (63.5)
(Q3.2) Use of medication	
Yes	15 (28.8)
No	37 (71.2)
(Q4) Vision or hearing impairment	
Yes	18 (34.6)
No	34 (65.4)
(Q5) Unsteady gait	
Yes	9 (17.3)
No	43 (82.7)
(Q6) Inadequate physical activity	
Yes	17 (32.7)
No	35 (67.3)

### Health conditions

The majority of participants (75%; *n* = 39) reported no history of falls in the past 12 months. More than half (63.5%; *n* = 33) were diagnosed with chronic diseases, 28.8% (*n* = 15) were taking medication, 34.6% (*n* = 18) had visual or hearing impairment, 17.3% (*n* = 9) had an unsteady gait, and 32.7% (*n* = 17) self-reported physical inactivity.

Correlation between the MIDT and physical measures. As presented in Table 3, a paired *t*-test revealed a significant correlation between the MIDT advanced program and the physical measures outcomes (*p* < 0.001).

**Table 3:** Paired *t*-test on mean values of physical measures (seconds) before and after the intervention.

Physical measures (in seconds)	Before intervention	After intervention	Paired differences 95% confidence interval	*p*-value	*t*-value	Effect size
Balance Rt_	21.6	41	9.866	*p* < 0.001	−3.205	−0.445
Balance Lt	22.5	39.7	8.765	*p* < 0.001	−2.320	−0.322
Mobility	10.07	9.57	0.41	*p* < 0.001	2.050	0.285

Balance Rt, right leg stands (seconds); Balance Lt, left leg stands (seconds).

Correlation between the MIDT and psychological measures. Table 4 summarizes the Wilcoxon test on the correlation between the MIDT program and the psychological measures of fear of falling and well-being. The mean difference in fear of falling scores was significantly lower in the pre-intervention group compared to the post-intervention measures (*p* < 0.01, *z* = 1.09), while the mean difference in well-being scores was significantly higher in the pre-intervention group than in the post-intervention group (*p* < 0.001, *z* = 0.3).

**Table 4:** Wilcoxon test on psychological measures before and after the intervention.

	Median differences between T1 and T2 groups for fear of falling	Median differences between T1 and T2 groups for the WHO-5
*Z*	1.0851	0.3094
Asymp. Sig. (two-tailed)	^**^p < 0.01	^***^p < 0.001

T1, before intervention; T2, after intervention. ^**^p < 0.01; ^***^p < 0.001.

## Discussion

The MIDT program engages participants in physical, psychological, and environmental interactions within a single dance therapeutics framework, rather than focusing solely on balance, strength, or mobility outcomes. It promotes an integrated approach to fall prevention practices among community-dwelling older adults. The increased sessions of core muscle strengthening, combined with integrated mindfulness techniques for managing fall risk behaviors in daily living environments, may contribute to a greater improvement in both physical condition and overall well-being (19). Meanwhile, it was observed that the more intense physical training and the increased dance therapeutics movements from the basic to the advanced program did not induce fear of falling or negatively affect the state of well-being, despite the greater range of mobility and dance movements.

### Postural adjustment and control

Most research on fall prevention focuses primarily on physical or muscle strengthening and balance, with relatively little attention given to intrinsic factors such as postural adjustment and fall efficacy. In addition to core muscle training, the body–mind therapy techniques in this advanced program, such as grounding and body centering, enhance self-awareness of body posture and movement, enabling participants to adjust and control postures in a group setting. These body–mind techniques may contribute to improved physical and psychological outcomes and specifically help prevent falls. It also addresses one of the prominent behavioral factors contributing to falls, which may not be targeted in normal physical training. Pitluk Barash et al. (23) reported that grounding techniques, commonly utilized in DMT, enhance physical awareness, improve sensory perception, and consequently promote a sense of security in a holistic manner. The body–mind intervention approach may change standing and walking habits and improve head–body–extremity coordination in older adults, thereby enhancing fall prevention.

### Behavioral factors affecting fall efficacy

Fall efficacy has been studied as a major factor contributing to falls in recent research (8). He et al. stated that “A fresh perspective of recognising falls efficacy as a perceived ability to manage a threat of a fall. Falls efficacy, with a broadened interpreted construct, relates to the individual’s perceived self-efficacy of performing necessary actions needed in different scenarios, including pre-fall, near-fall, fall-landing and completed fall” (13). [sic] For both basic and advanced programs, similar issues may be addressed through perturbation training using dance and improvisational movement. Pitluk Barash et al. (23) highlighted that interventions based on dance often incorporate key movement elements found in DMT, such as rhythm, synchronization, and spontaneity. This can serve as an effective simulation in a group setting to train reactions to falls and address related behavioral issues, making the advanced program more effective than training solely with technology or machines, which lack real human interaction.

Filar-Mierzwa et al. (14) examined dance therapeutics and balance in women over 60 years old. The study discovered that controlled weight shifting and ankle sway, along with alternating between a narrow and wide stance to continually change the base of support, helped participants improve their balance. Rotational trunk-driven movements, as well as dorsiflexion and plantar flexion during dance, may impact key sensorimotor elements, thereby enhancing movement recovery strategies and sensory integration.

### Environmental factors affecting fall efficacy

The World Health Organization (1) stated that the interplay between individuals’ physical condition and the surrounding environment affects the risk of falling. Most research, however, focuses primarily on changing the surrounding environment (15). Both intrinsic and extrinsic factors should be considered to prevent falls in older adults, resulting in better maintenance and reinforcement of their physical and psychological health in response to environmental changes.

### Perception and reaction to the environment and falls

Perception and reactions to the environment involve sensory awareness and the integration of sensory information with environmental cues. In a study by Pitluk Barash et al. (23), participants reported a heightened sense of security and control over themselves and surroundings after engaging in an integrated physical and dance movement therapeutics program. By enhancing perception and interaction with the environment, participants’ reaction to the environment and falls may be more sensitive and agile. Their interaction with themselves and the environment is different. The advanced dance therapeutics movements focus more on spontaneous and expressive motion, promoting sensory awareness and mind–body integration (22). Expressive movements that focus more on self-perception and on releasing fear through the self-expression of feelings and thoughts to the environment can increase environmental awareness, thereby improving the perception of fall risk.

### Interaction between individuals and the environment

Communicative movements, which focus on two-way communication in dance therapeutics, can contribute to optimizing the effects of the interaction between individuals and their environments. These communicative movements can enhance spatial awareness and distance perception through interactions with both human and non-human elements in the environment.

Communicative movements in response to changes in the perceived environment and reactions to falls are more individualized, which may require more one-to-one interaction to help individuals adjust and reposition themselves within the environment. While enhanced environmental perception is a contributing factor, the efficacy of MIDT may also stem from improvements in spatial cognition and spatial awareness, which influence how individuals orient themselves within a given space.

## Limitations

Several methodological limitations should be considered when interpreting the study results. First, the use of a single-group quasi-experimental design without a control group limits the ability to establish causal relationships between fall efficacy and prevention. In addition, the potential influence of the “Hawthorne effect” on participants’ self-reported outcomes could not be assessed, as participants were aware of being observed. This effect may have led to an inflation of the observed outcomes. Second, the small sample size resulted in limited statistical power, affecting the reliability of the findings and the ability to detect true effects. Third, the representativeness of the sample was limited by the high percentage of female participants. The uneven gender distribution among participants may reflect a higher interest in dance activities among female individuals. Future research will adopt a randomized controlled trial (RCT) design to improve gender balance and reduce sampling bias.

## Conclusion

This study provides a holistic perspective on fall intervention programs. Future research will employ a randomized controlled trial (RCT) design to control for confounding variables, reduce sampling bias, and establish the causal effects of the MIDT program on fall efficacy and prevention. A larger sample size will be required to improve statistical power and enhance the generalizability of the findings in relation to fall risk reduction. In addition, future studies should account for environmental factors, fear of falling, and state of well-being. Reactions to falls could be experienced more positively while maintaining a positive state of well-being. The integration of physical strengthening of the core and lower limb muscles with environmental perception and interaction may enhance the holistic state of well-being across physical, psychological, and environmental domains.

## Data Availability

The raw data supporting the conclusions of this article will be made available by the authors, without undue reservation.
